# Modeling immunoglobulin light chain amyloidosis in *Caenorhabditis elegans*

**DOI:** 10.1242/dmm.052230

**Published:** 2025-07-25

**Authors:** Margherita Romeo, Maria Monica Barzago, Alessandro Corbelli, Silvia Maglioni, Natascia Ventura, Carmina Natale, Andrea Conz, Mario Salmona, Giovanni Palladini, Mario Nuvolone, Fabio Fiordaliso, Giampaolo Merlini, Luisa Diomede

**Affiliations:** ^1^ Department of Molecular Biochemistry and Pharmacology, Istituto di Ricerche Farmacologiche Mario Negri IRCCS, 20156 Milan, Italy; ^2^ Institute of Clinical Chemistry and Laboratory Diagnostic, Medical Faculty, Heinrich Heine University, 40225 Düsseldorf, Germany; ^3^ Leibniz Research Institute for Environmental Medicine (IUF), 40225 Düsseldorf, Germany; ^4^ Department of Molecular Medicine, University of Pavia, 27100 Pavia, Italy; ^5^ Amyloidosis Research and Treatment Center, Fondazione IRCCS Policlinico San Matteo, 27100 Pavia, Italy

**Keywords:** Protein misfolding disease, Immunoglobulin light chain amyloidosis, Animal model, *C. elegans*, Pharynx

## Abstract

Cardiomyopathy determines the prognosis of patients with immunoglobulin light chain (AL) amyloidosis, a rare systemic disease caused by the misfolding and deposition of monoclonal light chains (LCs). The reasons underlying their cardiac tropism remain unknown, and an animal model recapitulating the main pathological features of AL amyloidosis is needed. Taking advantage of the similarities between the vertebrate cardiac muscle and *Caenorhabditis elegans* pharynx, we developed a new transgenic nematode expressing a human amyloidogenic λ LC, the sequence of which was deduced from a patient with AL amyloidosis with cardiac involvement (MNH). Strains expressing a non-amyloidogenic LC (MNM) or the empty vector only (MNV) were generated as controls. At variance with controls, LCs expressed in the body-wall muscle of MNH worms formed soluble dimeric assemblies, which could be secreted and reach different organs. Notably, MNH worms exerted a pharyngeal impairment resembling cardiac functional dysfunction in patients with AL amyloidosis, accompanied by increased radical oxygen species production and tissue ultrastructural damage. This new animal model could help to elucidate the mechanisms underlying the cardiac-specific toxicity occurring in AL amyloidosis, providing innovative insights into the pathophysiology.

## Teaser

**Summary:** We generated a new transgenic *C. elegans* strain as a model of immunoglobulin light chain amyloidosis to investigate the mechanisms underlying the cardiac-specific toxicity occurring in the disease and provide innovative insights into the pathophysiology.

## INTRODUCTION

Immunoglobulin light chain (AL) amyloidosis is a severe protein misfolding disease caused by a B-cell or a plasma cell clone, resulting in the overproduction of unstable monoclonal light chains (LCs) (Sabinot et al., 2023; Sanchorawala, 2006). After secretion into the bloodstream, LCs are transported to target organs, accumulating and exerting their toxic effects. Among the organs involved, the heart is one of the most frequently damaged and the one that causes the onset of rapidly progressive and fatal cardiomyopathies, determining patients' survival (Duca et al., 2023; Sanchorawala, 2024). Notably, cardiotoxicity is the leading cause of death in patients with AL amyloidosis, independently of the organs involved because ∼80-90% of patients die from heart failure or fatal arrhythmias (Lin et al., 2017; Merlini et al., 2018; Oghina et al., 2022; Ríos-Tamayo et al., 2023).

Today, the mechanisms underlying the tissue-specific cardiac targeting and damage of LCs are still undetermined, making it challenging to develop new pharmacological approaches to reduce the mortality of patients with AL amyloidosis (Bal and Landau, 2021; Bou Zerdan et al., 2023; Merlini et al., 2018; Palladini and Milani, 2023; Puri et al., 2023). Numerous attempts have been made to decipher the onset and progression of the AL amyloidosis pathological process, from amyloidogenic LC secretion to fibrillar deposit formation and organ tropism. Information on the general steps leading LCs to misfold and aggregate has been obtained from *in vitro* studies (Fishov et al., 2023; Kazman et al., 2021; Maritan et al., 2019; Oberti et al., 2019; Pradhan et al., 2023; Radamaker et al., 2021; Russo et al., 2022; Swuec et al., 2019). Experiments conducted with cardiomyocytes and cardiac fibroblasts provided advances in the mechanism of LC toxicity, showing that soluble oligomers and amyloidogenic deposits are both key players (Diomede et al., 2014, 2017; Kazman et al., 2021; Merlini, 2017; Zhang et al., 2023). Although soluble oligomers reduce cell viability through aberrant interactions with critical cellular components and generation of radical oxygen species (ROS), resulting in mitochondria damage (Diomede et al., 2017), the amyloid burden can contribute to organ damage by mechanical constraint and alteration of the tissue architecture (Merlini, 2017; Sanchorawala, 2024).

The efforts made over the years to develop adequate animal models of AL amyloidosis have also been particularly intense (Martinez-Rivas et al., 2022). In zebrafish, the injection of cardiotoxic LC resulted in cardiac dysfunction, cell death and early mortality (Duca et al., 2023; Mishra et al., 2019). Transgenic rodents bred to develop AL amyloidosis can produce free human amyloidogenic LCs (Ayala et al., 2021; Martinez-Rivas et al., 2022, 2024 preprint; Mishra et al., 2019; Nuvolone et al., 2017). These mice do not spontaneously develop amyloidosis; however, upon injection with fibrils or soluble forms of the variable domain of LCs, deposits appear, accompanied by cardiac dysfunction highly similar to that observed in humans (Martinez-Rivas et al., 2024 preprint).

The nematode *Caenorhabditis elegans* has become a major experimental organism with applications to many biomedical research areas. This model organism has spearheaded aging research and is widely used in experimental cardiology (Benian and Epstein, 2011) because its rhythmically pumping pharynx is considered an ortholog of the vertebrate cardiac muscle. The worm's pharynx is similar to the heart. It is a tubular muscular pump primarily composed of smooth muscle tissue and accessory neurons, capable of self-stimulation and autonomous generation of the depolarising signal independent of extra-organ neural stimulation (Avery and Shtonda, 2003; Haun et al., 1998; Jones and Candido, 1999; Mango, 2007; Nas, 2021). Contraction of the *C. elegans* pharynx is continuous, involuntary and persistent throughout the worm's entire life in a manner that is comparable to that of a more evolved heart (Haun et al., 1998; Nas, 2021). This model organism was earlier exploited to investigate the mechanism of cardiac toxicity by administering Bence Jones or recombinant LCs, demonstrating that only proteins from patients with amyloid cardiomyopathy damaged the pharynx of worms (Diomede et al., 2014).

In the present study, we developed a new *C. elegans*-based model of AL amyloidosis by generating a transgenic MNH strain constitutively expressing in the body-wall muscle cells the human amyloidogenic λ LC H7, the sequence of which was deduced from a patient with AL amyloidosis with cardiac involvement. To this end, we used the *mos1*-mediated single copy insertion (MosSCI) method to insert genetic cargo at defined locations in the worm's genome (Frøkjær-Jensen et al., 2008). Two additional strains were developed as controls: the MNM strain expressing the non-amyloidogenic λ LC M7, the sequence of which was deduced from a patient affected by multiple myeloma with no evidence of AL amyloidosis, and the MNV strain expressing the empty vector only.

Our findings indicated that the expression of the cardiotoxic amyloidogenic LC in the body-wall muscle of MNH worms generated a soluble dimeric protein, which can be secreted to reach various *C. elegans* organs. In MNH worms, we observed specific pharyngeal impairment, increased superoxide production and ultrastructural damage that did not occur in MNM and MNV worms.

These new *C. elegans* models offer a practical, informative approach for investigating the mechanisms underlying the tissue-specific toxicity of LCs. They could provide new insights into the pathophysiology of AL amyloidosis and represent a valuable tool for preclinical studies.

## RESULTS

### Generation of transgenic *C. elegans* strains

**Fig. 1.**
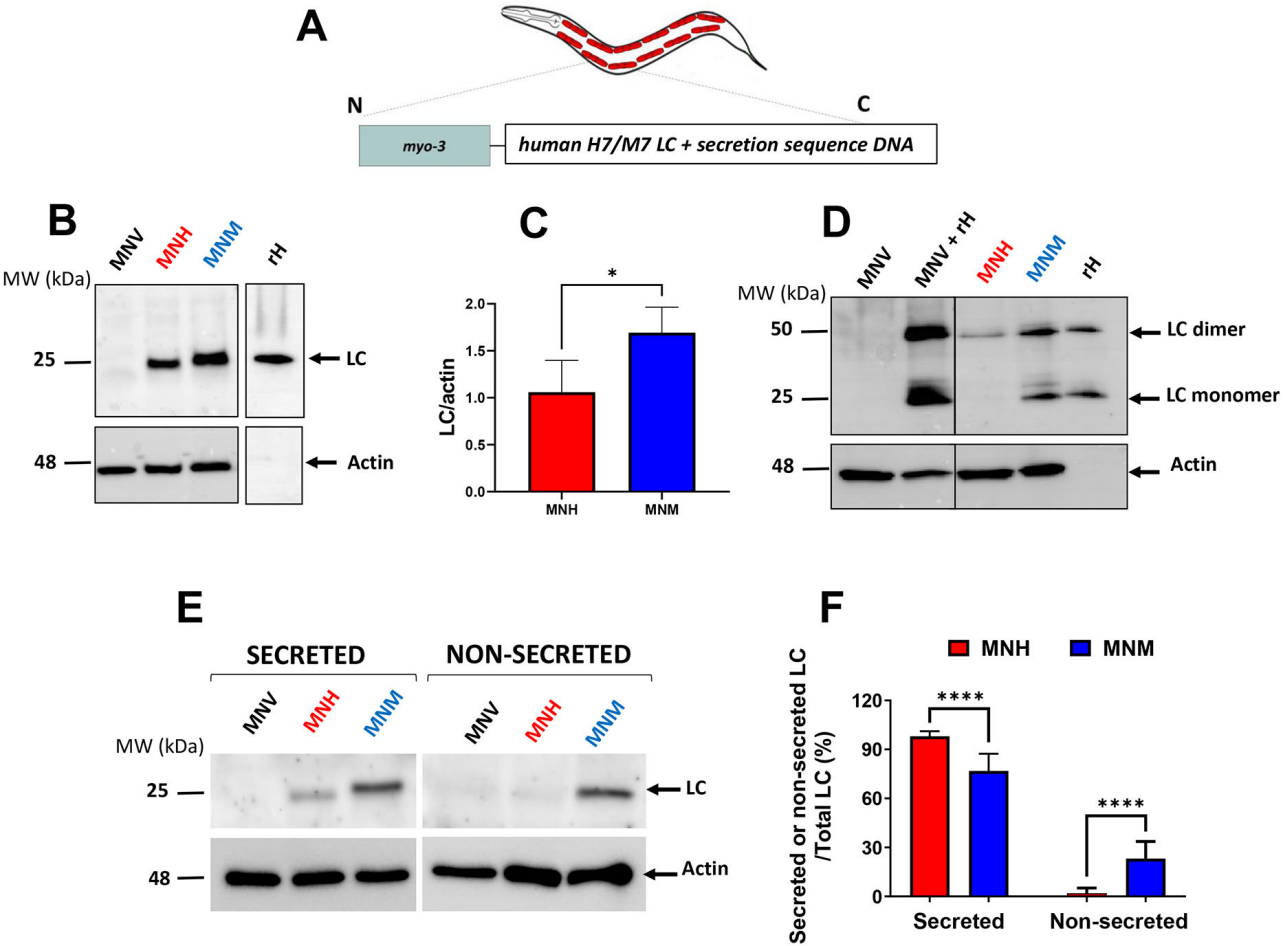
**Characterization of the transgenic *C. elegans* strains.** (A) Schematic representation of transgenic *C. elegans* transgenes generated in this study. The amyloidogenic cardiotoxic (H7) and non-amyloidogenic (M7) monoclonal light chains (LCs) were expressed under the body-wall muscle-specific promoter *myo-3* to generate MNH (constitutively expressing in the body-wall muscle cells the human amyloidogenic λ LC H7, the sequence of which was deduced from a patient with AL amyloidosis with cardiac involvement) and MNM (expressing a non-amyloidogenic LC) transgenic *C. elegans* strains. Human-specific secretion was included in each strain to enable LC to be secreted. (B,D) Western blots, representative of four biological replicates performed in reducing (B) and non-reducing (D) conditions of total LCs expressed by worms on the first day of adulthood. Equal amounts of proteins (25 µg) were loaded in each gel lane and immunoblotted with anti-human λ total LC or anti-actin antibody. Twenty-five ng of recombinant H7 LC (rH) was loaded alone, and 50 ng rH was loaded with 25 µg protein lysate of MNV worms as additional positive controls. (C) Quantification of total LCs expressed as the mean volume of the anti-human λ total LC band immunoreactivity of the western blot in B normalized to actin band. Data are mean±s.d. (*n*=4). (E) Representative western blots of secreted and non-secreted fractions of worms’ lysates on the first day of adulthood. Equal amounts of proteins (25 µg) were loaded in each gel lane and immunoblotted with anti-human λ total LC or anti-actin antibody. (F) Quantification of LC in secreted and non-secreted fractions is expressed as the percentage of total anti-human λ LC signal immunoreactivity in the secreted or non-secreted fraction/total LC (secreted+non-secreted) normalized to the actin immunoreactive signal (LC/actin) (mean±s.d., *n*=3). *P*<0.0001, unpaired, two-tailed Student's *t*-test. MW, molecular mass.

To develop a new animal model of AL amyloidosis, we generated a transgenic MNH *C. elegans* strain constitutively expressing the human amyloidogenic H7 LC with its specific secretion sequence deduced from a patient with AL amyloidosis with cardiac involvement under the body-wall muscle-specific promoter *myo-3* (Fig. 1A; Table S1, Fig. S1). A strain expressing a human non-amyloidogenic M7 LC and its secretion sequence deduced from a patient with multiple myeloma (MNM) (Fig. 1A; Table S1, Fig. S1) and a strain expressing the empty vector only (MNV) under the *myo-3* promoter were generated as controls. Transgenic worms were produced employing the MosSCI technique, which allows the insertion of a single copy of a transgene into chromosome II of the worm. We assessed the LC mRNA and protein levels expressed by the synchronized transgenic strains on the first day of adulthood. MNH worms expressed a significantly lower mRNA LC level than MNM worms; as expected, no signal was observed for MNV transgenic animals (Fig. S2). Accordingly, MNH worms produced a significantly lower amount of LC protein than MNM worms, as indicated by SDS-PAGE analysis under reducing conditions (Fig. 1B,C). No signal was detected in MNV worms (Fig. 1B), indicating that the antibody employed did not recognize any *C. elegans* endogenous proteins. Western blot analysis performed under non-reducing conditions showed that, in MNH worms, LCs are expressed only as dimers. In contrast, in MNM, the proteins are produced as monomers and dimers (Fig. 1D).

Amyloidogenic LCs are efficiently secreted by plasma cells and released into the bloodstream of patients with AL amyloidosis. To evaluate whether the LCs produced by MNH worms in the body-wall muscle cells can be released in the extracellular space too, we performed SDS-PAGE analysis of the supernatant and pellet obtained after centrifugation of homogenates of transgenic worms on the first day of adulthood in the absence of any detergent (Madhivanan et al., 2018) (Fig. 1E). Higher immunoreactive signals were observed in western blots obtained from the supernatant and pellet of MNM compared to MNH worms, confirming that the latter produced lower amounts of LCs (Fig. 1E). However, when the two fractions were calculated as percentage of the total LC produced, the amount of protein supernatant of MNM worms was significantly lower than that of the MNH worms (76.9±10.5% and 98.0±3.1% LC in the supernatant of MNM and MNH worms, respectively) (Fig. 1F). These data indicate that almost all the amyloidogenic LC produced by MNH worms can be released in the extracellular space. To confirm this finding, worms' lysates were sequentially fractionated into high-salt reassembly buffer (RAB) and detergent-soluble immunoprecipitation buffer (RIPA) to separate the soluble extracellular LC from those complexed with the membrane (Pir et al., 2016). The concentration of LCs found in the RAB of MNH worms was 5.07±0.59 ng/ml, whereas a significantly higher concentration was found in the RAB of MNM worms (9.70±1.64 ng/ml, *N*=5, *P*=0.0003, unpaired, two-tailed Student's *t*-test). Similar results, although semi-quantitative, were obtained by western blot analysis (Fig. S3A,B). These findings demonstrate that the lack of toxicity in the MNM strain cannot be attributed to the low concentration of non-amyloidogenic LCs in the extracellular medium.

**Fig. 2.**
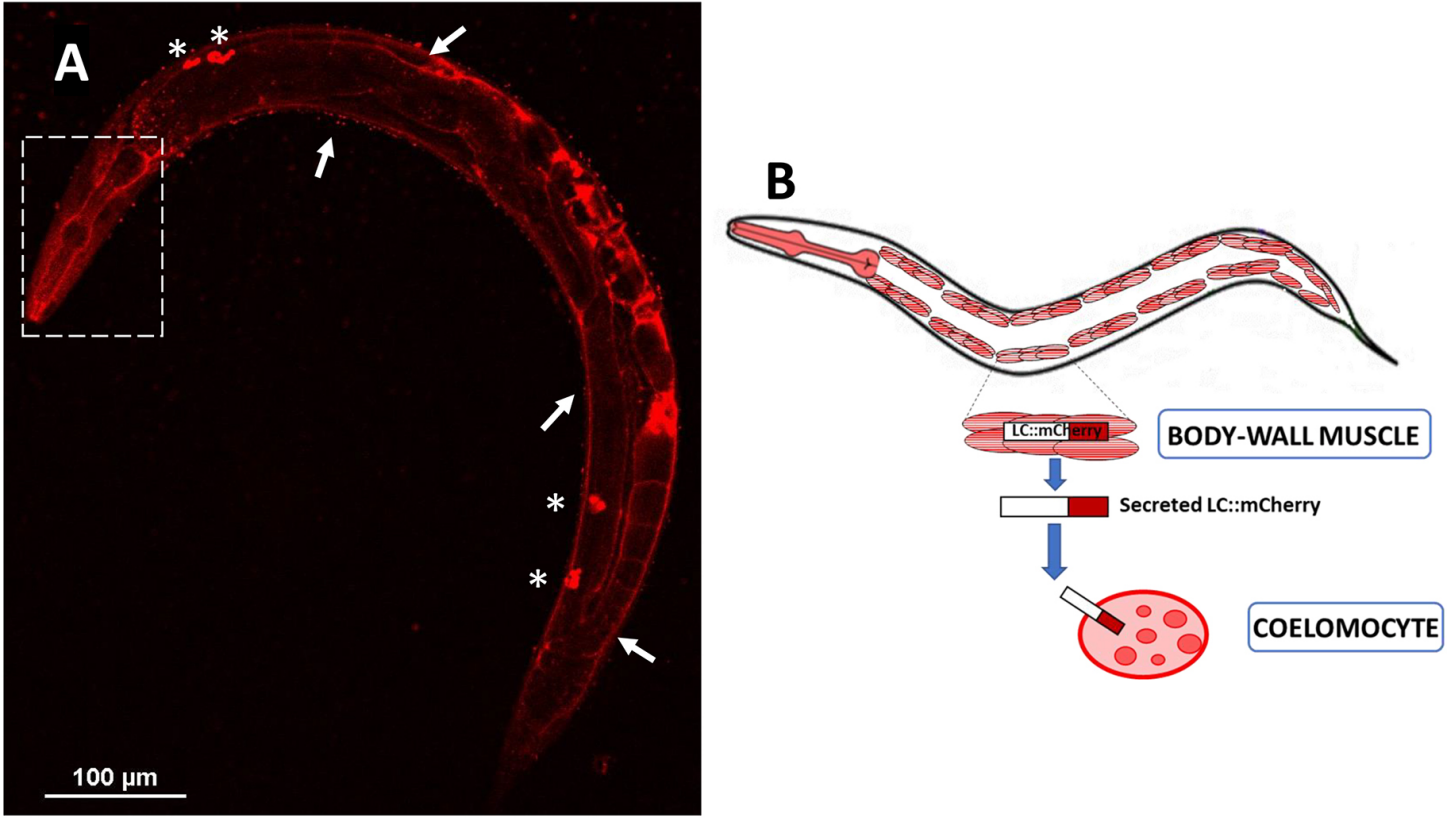
**Localization of the amyloidogenic LC in MNH::mCherry worms.** (A) Representative confocal image of MNH::mCherry worms on the first day of adulthood, showing a positive mCherry signal in the body-wall muscle (white arrows) and in coelomocytes (asterisks), demonstrating uptake of the secreted LC. A positive signal was also detected in the anterior region of the worm, including the pharynx (white dashed line box). (B) A picture representing LC-tagged synthesized in body-wall muscle cells by MNH::mCherry nematodes, their secretion pathway and coelomocyte uptake.

To follow the fate of the LCs produced by transgenic worms in the body-wall muscle cells, we planned to generate strains in which LCs were expressed and linked to the mCherry tag. Only worms expressing the amyloidogenic LC fused with mCherry were obtained (MNH::mCherry) because the expression of non-amyloidogenic protein fused with mCherry was not compatible with the survival of nematodes. Western blot analysis under reducing conditions indicated that transgenic MNH::mCherry worms, on the first day of adulthood, expressed an amount of LCs comparable to that produced by MNH nematodes of the same age (Fig. S4A,C) and exhibited similar pharyngeal dysfunction (Fig. S4D). In addition, western blot analysis performed under non-reducing conditions demonstrated that dimerization of the LC can also occur in this strain, although not completely (Fig. S4B). The incomplete dimerization may be due to the mCherry protein being fused with the LC at the C-terminus, where the covalent linkage of the disulfide bond occurs. Confocal analysis performed on MNH::mCherry worms indicated mCherry signal in the body-wall muscles and coelomocytes (Fig. 2A), specific cells with phagocytic activity (Tahseen, 2009). This demonstrates that after LCs are synthesized in body-wall muscle cells, they are released in the extracellular space, reaching the body fluid and then taken up by coelomocytes, accumulating in large vesicles (Egami et al., 2014) (Fig. 2B). Noteworthily, amyloidogenic LCs can also reach other organs, particularly the pharynx of worms (Fig. 2A).

Taken together, these data show that expression of a cardiotoxic amyloidogenic LC in the body-wall muscle of worms generated a soluble dimeric protein that can be released and reach the *C. elegans* ‘ancestral heart’ – the pharynx.

### Expression of the amyloidogenic LC in MNH worms causes a specific pharyngeal dysfunction and structural damage

**Fig. 3.**
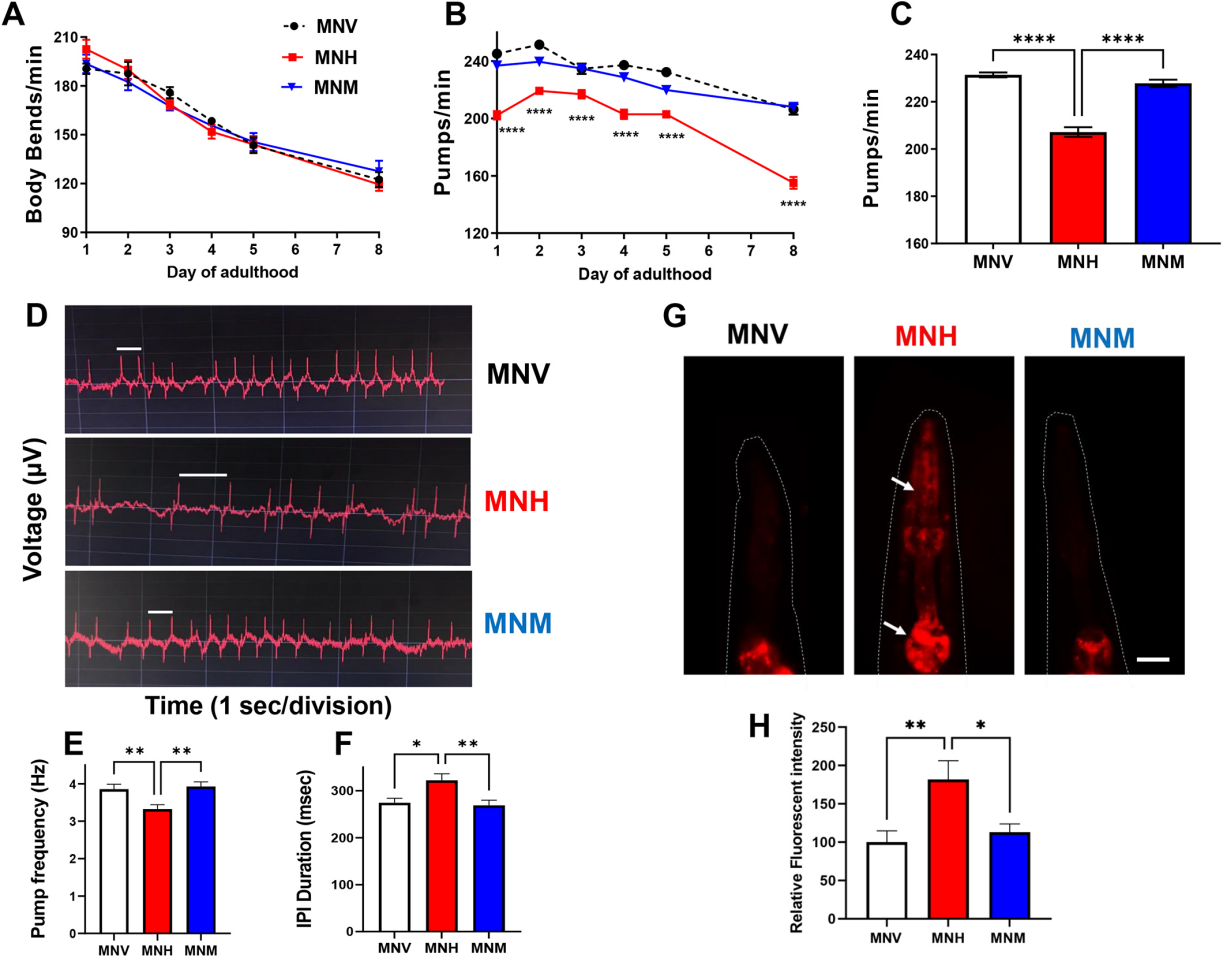
**Expression of human cardiotoxic LC in MNH transgenic worms induced specific pharyngeal impairment.** (A,B) Transgenic worms' motility (A) and pharyngeal activity (B) from the first to the eighth day of adulthood. Data are expressed as the mean of body bends/min (A) or pumps/min±s.e.m. (B) (*n*=20 worms/assay, three assays). ****P<0.0001 versus the corresponding MNV and MNH time point, one-way ANOVA and Bonferroni's post hoc test. (C) The pharyngeal activity of worms on the first day of adulthood is expressed as pumps/min. Data are mean±s.e.m. (*n*=40 worms/assay, four assays). ****P<0.0001, one-way ANOVA and Bonferroni's post hoc test. (D) Representative electropharyngeograms of worms on the first day of adulthood. The voltage change was plotted against time. A positive voltage change can be observed as an excitatory spike upon pharynx contraction. The relaxation of the pharynx muscle resulted in an adverse voltage change, the relaxation spike. (E) The pump frequency was expressed in Hz. (F) The interpump interval (IPI; i.e. the time between two excitatory spikes, white lines in D) was defined in ms. (E,F) Data are mean±s.e.m. (*n*=30 worms/assay, three assays). *P<0.001 and **P<0.005, one-way ANOVA and Tukey's post hoc test. (G) Representative images of mitochondrial superoxide production in the pharynx of worms, detected with MitoSOX™ Red. Indicated by red fluorescence, an increase in intracellular superoxide production was observed in MNH worms' anterior and terminal pharyngeal bulbs (arrows). Scale bar: 50 µm. (H) Relative quantification of MitoSOX™ Red fluorescence intensity measured by ImageJ software. Data are mean±s.e.m. (*n*=30 worms/assay, three assays). *P<0.001 and **P<0.005, one-way ANOVA and Bonferroni's post hoc test.

Experiments were performed to investigate whether the expression of the amyloidogenic LC in MNH worms translated to the onset of specific phenotypic dysfunctions. To this end, various behavioral tests were performed on MNH, MNM and MNV nematodes. We measured the neuromuscular activity of the transgenic worms by counting the number of movements in a liquid and their pharyngeal motility by scoring the number of pharyngeal contractions in 1 min (Fig. 3A,B).

In MNH worms, from the first to the eighth day of adulthood, we observed a physiological decline in neuromuscular activity similar to that in the MNM and MNV control strains (Fig. 3A), indicating that, in young/adult worms, the expression of the amyloidogenic LC in the body-wall muscle cells did not cause any specific neuromuscular defect. Similar data were obtained from the evaluation of worms' healthy aging by scoring the ability of animals to crawl spontaneously or after a manual stimulus (healthspan) (Fig. S5A). However, with increase in worm age, specifically from day 12, the amyloidogenic and non-amyloidogenic LC expression reduced the ability of MNH and MNM worms to move spontaneously compared to MNV worms (Fig. S5A) and caused a significant reduction in the median healthspan and lifespan of the animals (Table S2, Fig. S5B). These findings indicate that amyloidogenic and non-amyloidogenic LC production impaired the worms' muscular function during aging.

In MNH nematodes, we observed significant impairment of pharyngeal function, which worsened with age (Fig. 3B). This defect was explicitly related to the expression of amyloidogenic LC because no pharyngeal impairment occurred in MNM and MNV worms, in which only a physiological age-dependent decline was observed (Fig. 3B). On the first day of adulthood, the pharyngeal activity of MNH worms was ∼15% lower than that of MNV and MNM worms (Fig. 3C) and became ∼24% lower at day 8 (Fig. 3B). In addition, electropharyngeograms registered in worms on day 1 of adulthood indicated that MNH worms exhibited a significantly reduced pharyngeal pump frequency and an increase in the interpump interval (IPI; i.e. the interval between an excitatory spike and the following relaxation spike) compared to MNM and MNV worms (Fig. 3D-F). These data confirmed that the amyloidogenic LC responsible for the onset of amyloidosis with cardiac involvement in patients with AL amyloidosis caused a pharyngeal-specific dysfunction when expressed in *C. elegans*. One of the mechanisms underlying the proteotoxicity of amyloidogenic LCs is their ability to increase ROS production, particularly in mitochondria, which we have documented in the *C. elegans* model (Diomede et al., 2014, 2017). To evaluate whether the pharyngeal dysfunction in MNH worms was linked to superoxide production, nematodes were fed with MitoSOX™ Red, which is able to permeate live cells, selectively targeting mitochondria. As shown in Fig. 3G,H, in the pharynx of MNH worms, there was a strong increase in the fluorescent red signal compared to that in the pharynx of MNV and MNM worms, indicative of a significant increase in superoxide production in the mitochondria of animals expressing the amyloidogenic LC. The fluorescent signal observed in the MNH pharynx was similar to that caused in MNV pharynx by administering exogenous H_2_O_2_, which is used as a chemical stressor (Fig. S6). No fluorescent red signal was observed in the body-wall muscles of the worms of the three strains, indicating that the pharyngeal increase in superoxide production in MNH worms is tissue specific (Fig. S7).

**Fig. 4.**
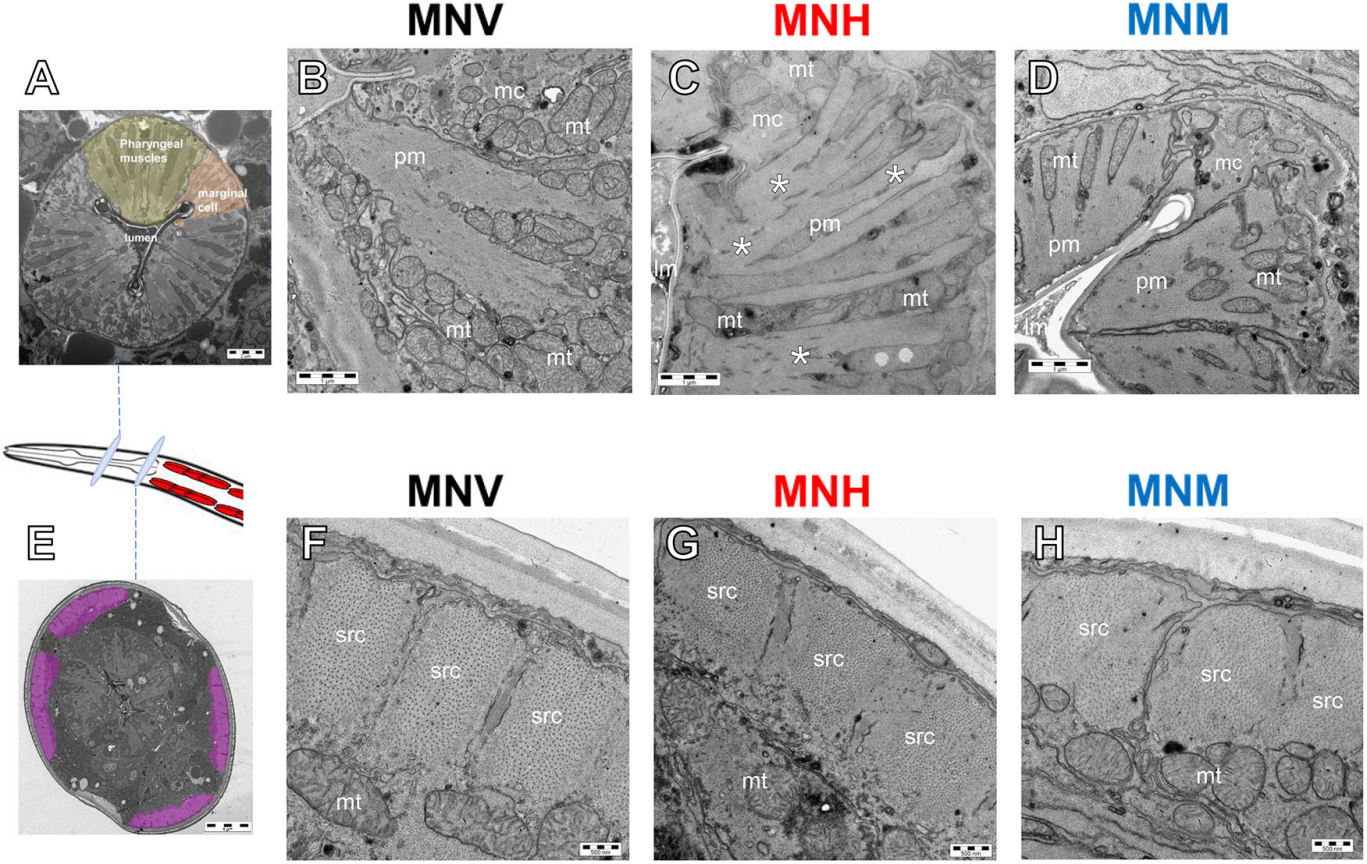
**The expression of cardiotoxic LC in MNH worms severely affects pharyngeal ultrastructure.** (A-H) Representative images of a worm's pharynx (A-D) and body-wall muscles (E-H) obtained from the ultrastructural analysis by transmission electron microscopy (TEM) in the different strains. A transverse section at low magnification above the second bulb of the pharynx shows the characteristic pharyngeal structure with pharyngeal muscles (pm; in green) separated by a marginal cell (mc; in orange), placed at the corner of the pharyngeal lumen (lm) (A), also observed at higher magnification in the pharynx of MNV (B) and MNM (D) worms. Conversely, the MNH (C) pharyngeal ultrastructure was seriously compromised by the presence of amorphous material (asterisks) in place of the radially oriented contractile filaments in pharyngeal muscles of MNV and MNM worms. Mitochondrial (mt) internal components in pharyngeal muscles and marginal cells were damaged. A transverse section of body-wall muscles (in purple) at low magnification (E). No ultrastructural damage of longitudinally oriented myofilaments of sarcomeres (src) or mitochondria was observed in the body-wall muscles of MNM (H) and MNH (G) compared to MNV (F) worms.

Ultrastructural studies were then performed to evaluate whether pharyngeal dysfunction can also be linked to alterations in the organ subcellular compartments, particularly mitochondria, which play a vital role in providing energy for contractile activity. In particular, transmission electron microscopy (TEM) studies were performed on the transverse sections of transgenic animals on the first day of adulthood to analyze the pharynx (Fig. 4A-D) and body-wall muscles (Fig. 4E-H). Profound ultrastructural alterations of contractile apparatus were observed in the pharynx of MNH worms, with disruption of the contractile filaments in pharyngeal muscles and mitochondrial damage in pharyngeal muscles and marginal cells (Fig. 4C), but not in the pharynx of MNM worms (Fig. 4D), the morphology of which was comparable to that of MNV pharynx (Fig. 4B). No ultrastructural damage to myofilaments or mitochondria was observed at this age in the body-wall muscles of MNH (Fig. 4G) and MNM worms (Fig. 4H), compared to those of MNV worms (Fig. 4F), even though LCs are expressed in this tissue compartment.

To investigate whether the pharyngeal dysfunction can be related to the formation of amyloidogenic LC deposits, X-34, a highly fluorescent derivative of Congo Red (Styren et al., 2000), was administered to MNH and MNH::mCherry worms from the first to the fifth day of adulthood (Link et al., 2001). No X-34-positive deposits were observed in the pharynx or the body-wall muscle at any of the ages considered (data available upon request), indicating that the pharyngeal-specific toxicity in MNH worms can be ascribed to the presence of soluble LC conformers in the tissue.

### MNH strain can be used for preclinical studies

**Fig. 5.**
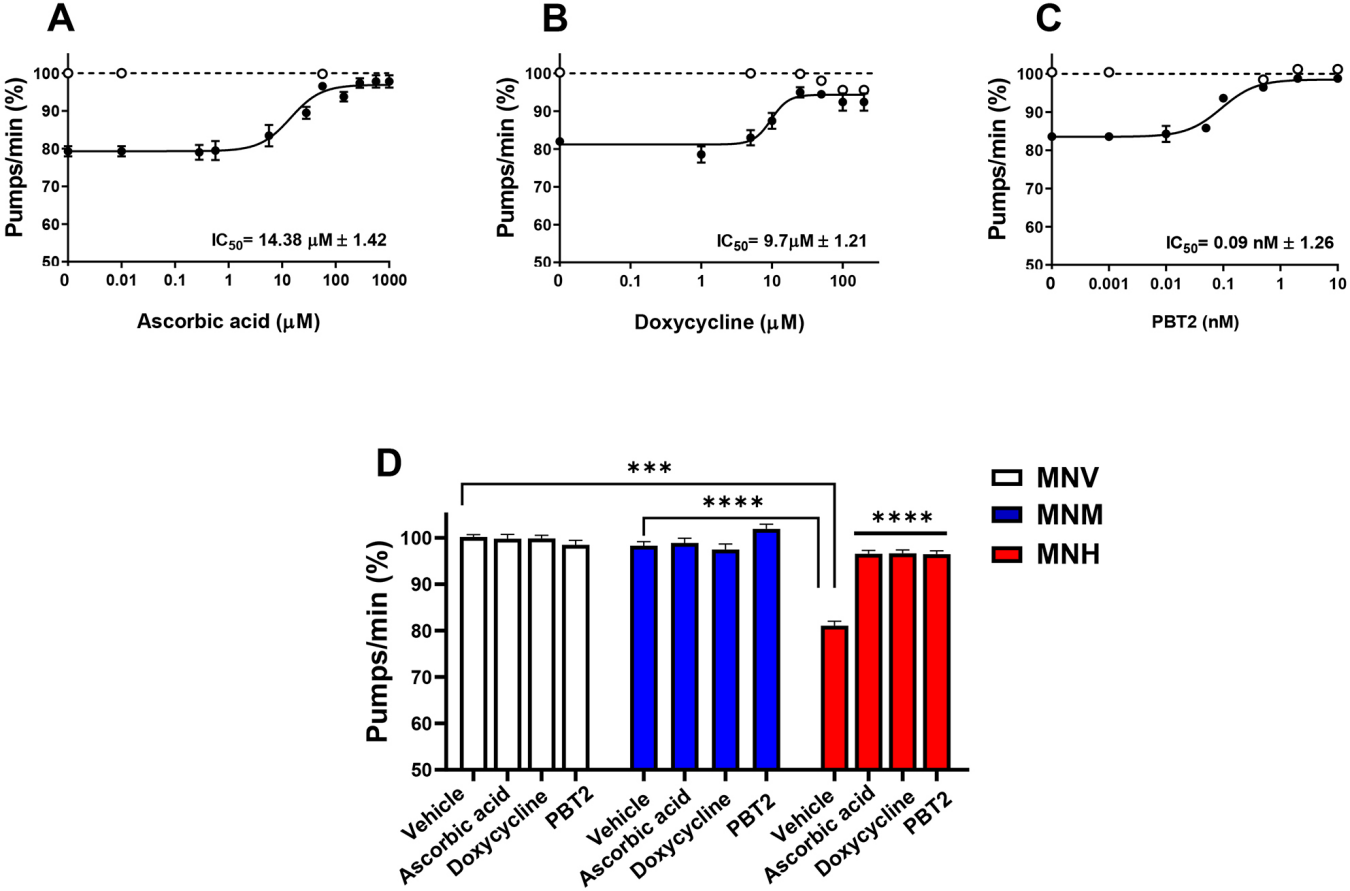
**Use of the MNH worms for preclinical studies.** (A-C) Dose–response effect of ascorbic acid (A), doxycycline (B) and PBT2 (C) on pharyngeal dysfunction in MNH worms. MNV (open circles) and MNH (filled circles) worms on the first day of adulthood were treated for 2 h at room temperature with increasing drug concentrations (100 worms/100 µl). Control MNV worms were treated with 10 mM PBS, pH 7.4 (dashed lines). Worms were then plated on NGM seeded with *E. coli*, and the pumping rate was scored after 24 h. Data were normalized to those of MNV control worms for each experiment and expressed as the mean percentage of pumps/min±s.e.m. (*n*=30 worms/group, three assays). Half maximal inhibitory concentration (IC_50_) values±s.e.m. are reported. (D) Worms were treated for 2 h at room temperature with 57 µM ascorbic acid, 25 µM doxycycline or 0.5 nM PBT2 (100 worms/100 µl). Control worms were treated with 10 mM PBS, pH 7.4 (vehicle). Worms were then plated on NGM seeded with *E. coli*, and the pumping rate was scored after 24 h. Data were normalized to those of MNV control worms for each experiment and expressed as the mean percentage of pumps/min±s.e.m. ***P<0.0005 and ****P<0.0001, two-way ANOVA and Bonferroni's post hoc test. Interaction MNH/MNH+ascorbic acid=*P*<0.0001, MNH/MNH+doxycycline=*P*<0.0001, MNH/MNH+PBT2=*P*<0.0001.

We also performed some experiments to explore whether the MNH strain can be used as an animal model of AL amyloidosis to discover and test novel pharmacological treatments. To this end, we selected ascorbic acid as the prototypic antioxidant already demonstrated to revert the toxicity of cardiotoxic LCs administered to worms owing to its ability to counteract ROS-induced pharyngeal damage (Diomede et al., 2017). We also used doxycycline, which reduced LC aggregation in a transgenic mouse model of AL amyloidosis and the toxicity of LCs administered to *C. elegans* (Diomede et al., 2014; Ward et al., 2011). Based on our previous findings demonstrating the pivotal role of metal ions, particularly copper, in LC-induced toxicity (Diomede et al., 2017; Russo et al., 2022), PBT2, an 8-hydroxyquinoline derivative acting as copper/zinc ionophore, was used as a metal-chelating compound (Summers et al., 2022). When administered alone, PBT2 permanently blocks ROS production and prevents the toxic effects caused in *C. elegans* by feeding amyloid LCs (Diomede et al., 2017). We observed that a single administration of all the drugs tested reduced, in a dose-dependent manner, the pharyngeal dysfunction of MNH worms on the first day of adulthood (Fig. 5A-C). Half maximal inhibitory concentration (IC_50_) values in the same order of magnitude were obtained for ascorbic acid (14.38±1.42 µM) and doxycycline (9.70±1.21 µM) (Fig. 5A,B). Starting from 50 µM, doxycycline became toxic, as indicated by its ability to significantly reduce pharyngeal function in MNV worms (Fig. 5B). PBT2 was the most effective compound, with an IC_50_ value of 0.09±1.1.26 nM (from 108,000- to 160,000-fold more effective than the other drugs tested) (Fig. 5C). When administered to MNH worms at their optimal concentration, ascorbic acid (57 µM), doxycycline (25 µM) and PBT2 (0.5 nM) allowed the full recovery of the pharyngeal defect caused by the amyloidogenic LC expression (Fig. 5D). Although the exact mechanism by which these pharmacological treatments subverted the pharyngeal dysfunction is not known, we can hypothesize that, similarly to what occurs when they are administered together with cardiotoxic amyloidogenic LCs, they exert multiple mechanisms of action involving both their simple antioxidant effect and their ability to act as metal chaperones to promote protective intracellular signaling by transporting metals into the cells (Diomede et al., 2017). These findings indicate that this strain can provide a useful tool for investigating the efficacy of drugs in protecting against LC-induced tissue damage.

## DISCUSSION

We herein generated and characterized the first transgenic *C. elegans* model expressing a human amyloidogenic LC in which the pharyngeal dysfunction resembled the cardiac-specific dysfunction observed in patients with AL amyloidosis. In particular, we expressed a λ LC, representing the most common isotype responsible for ∼80% of AL amyloidosis cases (Desport et al., 2012).

The amyloidogenic LCs expressed in the muscle cells of MNH worms were almost wholly released in the extracellular space. They reached the pharynx, causing a specific toxic effect similar to that in patients with AL amyloidosis whereby LCs, secreted into the bloodstream by bone marrow plasma cells, cause cardiotoxicity and progressive severe cardiac dysfunction. No functional and structural damage was observed in the body-wall muscle tissue in which LCs were produced, at least in the first week of adulthood. Expression of amyloidogenic LC impaired MNH worms' healthy aging processes and reduced their survival. However, a similar effect also occurred in MNH worms, indicating a general toxic effect of LC expression in the body-wall muscle cells of *C. elegans*.

Interestingly, the expression in MNH worms of the H7 amyloidogenic LC caused a pharyngeal defect comparable to that observed in our previous studies when the same LC was administered to wild-type *C. elegans* (Diomede et al., 2014, 2017). Moreover, in the pharynx of MNH worms and in worms fed H7, there is also increased production of mitochondrial ROS and marked structural damage, resembling that found on autopsy examination of the hearts of patients with AL amyloidosis (Diomede et al., 2017).

In MNM worms generated as an additional control to demonstrate the specific effect of amyloidogenic LC expression, although LCs were secreted and present in the extracellular medium at a higher concentration than that of amyloidogenic LCs, no structural damage was noted in the pharynx and muscle tissue of young worms. Reduced lifespan and healthspan with increasing age of the worms, also observed in MNH worms, was identified, indicating a general toxic effect of LC expression in the body-wall muscle cells of *C. elegans*.

We observed that the mRNA and protein levels of the non-amyloidogenic LC in MNM worms were about twice that of the amyloidogenic LC expressed by MNH worms. This is consistent with the quality control system's role in reducing the production of the misfolded protein (Diomede et al., 2012). Amyloidogenic LCs that escape the quality control mechanism may form unstable/misfolded dimers that exert a toxic *in vivo* effect in *C. elegans*.

We have not found amyloid fibrils in the pharynx or the body-wall muscle in MNH worms. Based on the knowledge that protein aggregation increases with age in *C. elegans* and with the temperature to which they are exposed by regulating protein homeostasis (Cuanalo-Contreras et al., 2022; Lee et al., 2023) and proteasome activity (Lee et al., 2023), several experiments were done on worms from the first until the ninth day of adulthood, cultivating them at 20°C or 25°C. We could observe, at most, in adult worms on the ninth day of adulthood maintained at 25°C, an increase in the size of mCherry-positive spots in MNH::mCherry worms in which X-34 positivity was not always detectable. Given that the staining of the amyloid fibers was done by feeding the worms X-34, owing to the reduced pharyngeal functionality of MNH nematodes, the concentration of dye that arrived in conjunction with the LCs could not always be sufficient to give a specific signal. TEM analysis could not detect the presence of amyloid fibrils in the pharynx and body-wall muscle of MNH transgenic animals on the first day of adulthood. We cannot exclude that the formation of amyloid deposits occurred beyond the tenth day of adulthood when worms began to die, and their pharyngeal function was so compromised as to not allow *in vivo* staining with X-34.

To gain insight into the pharyngeal dysfunction of MNH worms, we registered the electropharyngeograms (i.e. the pharyngeal electrical activity), which provide information on parameters such as the rate of pharyngeal pumping and the duration of each pump (Raizen and Avery, 1994). We observed that the pharynx contracted less frequently in MNH worms than in control worms, possibly reflecting electrical disturbances and functional impairment in the hearts of patients with AL amyloidosis (Falk et al., 2016). Although the circuitry of the pharynx is similar to that of the heart, with gap junctions coupling and synchronizing myocytes for potential conduction (Mango, 2007), it may be taken into account that many structural differences exist between proteins involved in generating potential (Raizen and Avery, 1994). Despite its limitations, the functionality of the nematode pharynx, similar to the mammalian cardiac muscle, has been shown to decline with age (Eckers et al., 2016) and in *C. elegans* models of mitochondrial diseases (Maglioni et al., 2022), which often present with cardiomyopathy in humans. Although the MNH transgenic worms described in our work represent a straightforward, low-cost animal model to rapidly study the mechanisms underlying cardiac toxicity of LC in AL amyloidosis, they are not without limitations. In particular, the mechanisms driving the cardiac tropism involve toxicity and the preferential localization of the LC, which is difficult to prove in *C. elegans* owing to the absence of other human surrogate organs, such as the kidney or liver, that AL amyloidosis can target. In addition, *C. elegans*, although formed by specialized cells, lacks some of mammals' anatomical features, including a vascular system and a blood–brain barrier. However, the distance of the anatomy of this nematode from that of vertebrates and humans does not prevent the use of *C. elegans* to model pathological conditions, particularly in the case of human pathologies such as rare diseases. We also know that a strain expressing an amyloidogenic non-cardiotoxic LC will be informative and improve this study. For future studies, we will consider generating a strain expressing an amyloidogenic non-cardiotoxic LC. Additional studies, such as single-cell RNA sequencing, will help us understand whether organ tropism is controlled by specific genes/proteins or whether other mechanisms are involved.

As indicated by the data obtained with the prototypical drugs we used in the study, MNH worms also represent an excellent model to evaluate the preclinical efficacy of new molecules. They offer a significant contribution to studies that could only be carried out *in vitro*, in cellular models, and using purified LCs.

## MATERIALS AND METHODS

### Transgenic *C. elegans* strains

All *C. elegans* strains were cultured and handled using standard breeding conditions. Experiments were performed at 20°C on standard nematode growth medium (NGM) seeded with OP50 *Escherichia coli* (*Caenorhabditis* Genetics Center, Minneapolis, MN, USA). To model AL amyloidosis, a new transgenic *C. elegans* strain was generated by InVivo Biosystems (Eugene, OR, USA) through the MosSCI method, allowing the insertion of a single copy of a transgene into chromosome II of the worm (Frøkjær-Jensen et al., 2008). The *C. elegans* strain was engineered to express the human amyloidogenic λ LC H7 constitutively in the body-wall muscle cells under the *myo-3* promoter (MNH) (Diomede et al., 2014) (Table S3). The H7 sequence inserted was deduced from the cDNA isolated from the bone marrow cells of a patient with AL amyloidosis with cardiac involvement (Perfetti et al., 1996). As controls, a strain constitutively expressing under the *myo-3* promoter the human non-amyloidogenic LC M7, the sequence of which was deduced from the cDNA of a patient with multiple myeloma (Garofalo et al., 2021) (MNM), and a strain expressing the empty vector alone (MNV) were developed (Table S3). The two LCs, H7 and M7, were expressed in worms with their specific human secretion sequences (ACCTGCTCCCCTCTCCTCCTCACCCTCCTTATTCACTGCACCGGATCCTGGACC and GCTTGGACTCCTTTATGGCTCACTCTCCTTACGCTGTGCATCGGATCCGTCGTC DNA sequences for H7 and M7, respectively). The full-length H7 and M7 protein sequences are reported in Fig. S1. Transgenic selection was done by screening strains by PCR by InVivo Biosystems for single-copy insertion at the *mos1* locus. A further transgenic strain was generated by InVivo Biosystems inserting the mCherry DNA sequence at the C-terminus of the H7 sequence expressed by MNH using CRISPR-site direct mutagenesis (MNH::mCherry) (Table S3). The relative levels of H7 and M7 genes expressed by MNH and MNM worms were determined by quantitative PCR (Q-PCR) (Table S4).

### mRNA extraction, PCR and Q-PCR

The nematodes were synchronized by egg laying and cultured on NGM plates seeded with OP50 *E. coli* for food at 20°C. On the first day of adulthood, nematodes were collected with M9 buffer, settled by gravity and washed with M9 buffer to eliminate bacteria. According to the manufacturer's instructions, RNA was extracted from the pellet of worms using a Maxwell^®^ RSC simplyRNA Tissue Kit (Promega Italia, Milan, Italy). Briefly, the pellet of worms was homogenized by Turrax (T10, IKA-Werke, Staufen im Breisgau, Germany) for 2 min using 200 μl of a chilled working solution prepared by adding 20 μl 1-thioglycerol per milliliter of homogenization solution. Lysis buffer (200 μl) was added to 200 μl homogenate, vortexed for 15 s and transferred into the cartridge well. RNA was extracted and eluted in 50 μl nuclease-free water, and the concentration was quantified using a NanoDrop spectrophotometer (Thermo Fisher Scientific, Monza, Italy). mRNA was reverse transcribed using a FIREScript RT cDNA Synthesis Kit (Solis BioDyne, Tartu, Estonia). To this end, 1 µg RNA was retrotranscripted using oligo(dT) primer in a 20 µl final mix volume. cDNA (50 ng) was used for Q-PCR amplification using 2X Power SYBR™ Green PCR Master Mix (Applied Biosystems, Thermo Fisher Scientific, Waltham, MA, USA) and an Applied Biosystems 7300 Real-Time PCR System. Two specific primers were designed on the constant region of H7 and M7 sequences and were used for the PCR amplification (Table S4). The relative level of H7 and M7 genes expressed by MNH and MNM worms was determined using the 2−ΔCT method using the cell division cycle-related (*cdc-42*) and the conserved iron binding-related (*y45f10d.4*) genes as housekeeping genes (Table S4).

### Western blot analysis

The nematodes were synchronized and cultured on NGM plates seeded with OP50 *E. coli* for food at 20°C. On the first day of adulthood, nematodes were collected in M9 buffer, settled by gravity and washed with M9 buffer to eliminate bacteria. To evaluate the total LC protein levels, pellets from 50 worms were suspended in 50 μl 1× SDS loading sample buffer (10% glycerol, 2% SDS, 60 mM Tris-HCl, pH 6.8), and 5% β-mercaptoethanol was added. Samples were boiled for 10 min at 95°C, and 25 μl of the samples was loaded into 12% SDS-PAGE gel wells. Recombinant H7 protein (50 ng), kindly provided by Professor Stefano Ricagno (University of Milan, Milan, Italy), was loaded as a control. To perform western blot analysis under non-reducing conditions, the worm pellets were suspended in 200 μl cold 10 mM PBS supplemented with complete protease inhibitors (Roche, Basel, Switzerland) and homogenized in ice by Turrax homogenization for 2 min. Samples were spun at 5900 ***g*** for 5 min at 4°C, the supernatant was collected, and the protein concentration was determined using a Pierce BCA Protein Assay Kit (Thermo Fisher Scientific). Samples (25 μg total protein) were suspended in 1× SDS loading sample buffer without β-mercaptoethanol, boiled for 5 min at 95°C and loaded into the wells of a 12% SDS-PAGE gel. Recombinant H7 (50 ng) was loaded alone or with 25 µg protein lysate of MNV worms as positive controls.

To quantify secreted and non-secreted LCs, the worm pellets were suspended in 200 μl cold 10 mM PBS supplemented with complete protease inhibitors (Roche) and homogenized in ice by Turrax homogenization for 2 min. Samples were spun at 3000 ***g*** for 15 min at 4°C, and the supernatant was collected as the fraction containing secreted LCs. The pellet, collected as the fraction containing the non-secreted LCs (Madhivanan et al., 2018), was washed twice with 10 mM PBS, re-suspended in a hot 10% SDS solution and boiled for 10 min at 95°C. The protein concentrations of secreted and non-secreted fractions were determined using the Pierce BCA Protein Assay Kit. Twenty-five μg of total proteins were suspended in 1× SDS loading sample buffer containing 5% β-mercaptoethanol, boiled for 5 min at 95°C and loaded into the wells of a 15% SDS-PAGE gel.

To evaluate LC solubility, proteins were extracted from transgenic worms on the first day of adulthood using RAB [100 mM 2-(N-morpholino) ethanesulfonic acid, 1 mM EGTA, 0.5 mM MgSO_4_, 20 mM NaF] and RIPA [150 mM NaCl, 1% (v/v) Nonidet P-40, 0.5% (w/v) deoxycholate, 0.1% SDS, 50 mM Tris-HCl, pH 8.0] buffers containing EDTA-free protease inhibitors as previously described (Morelli et al., 2018). The concentration of LCs in the RAB fraction was determined using a human lambda immunoglobulin light chain (λ-IgLC) enzyme-linked immunosorbent assay kit (MBS263093, DBA Italia, Milan, Italy), following the manufacturer's instructions. Data were expressed as the mean concentration±s.d., subtracted from the blank value.

Proteins in all the SDS-PAGE gels were separated at 100 V in SDS running buffer and blotted for 2 h at 100 V onto a PVDF membrane in 20 mM Tris solution containing 150 mM glycine and 10% methanol. Membranes were blocked for 1 h at room temperature in 10 mM Tris-HCl solution, pH 7.5, containing 100 mM NaCl, 0.1% (v/v) Tween 20, 5% (w/v) low-fat dry milk powder and 2% (w/v) bovine serum albumin, and incubated overnight at 4°C with a mouse monoclonal anti-human λ light chains (bound and free) antibody (1:1000; L6522, Sigma-Aldrich, Milan, Italy), or a mouse monoclonal anti-actin antibody clone C4 (1:2000; MAB1501, Sigma-Aldrich). Anti-mouse IgG peroxidase conjugate (1:10,000; A4416, Sigma-Aldrich) was used as the secondary antibody. Chemioluminescence was detected by Clarity Max Western ECL Substrate (Bio-Rad, Hercules, CA, USA), and the membranes were scanned with a ChemiDoc Imaging System (Bio-Rad). The mean volumes of immunoreactive bands were determined using Image Lab™ software (Bio-Rad).

### Lifespan and healthspan

Experiments were performed at 20°C on standard NGM seeded with OP50 *E. coli*. The nematodes were synchronized by egg laying and transferred to fresh NGM plates daily during the fertile period to avoid overlapping generations. Dead, alive and censored animals were scored during the transferring process. The animals were counted as dead when they had neither moved nor reacted to a manual stimulus with a platinum wire, nor had any pharyngeal pumping activity. Animals with exploded vulvas or those desiccated on the wall were censored (Leiser et al., 2016). The number of active movements was also assessed in nematodes employed for the lifespan assay to determine their healthy aging. Animals crawling spontaneously or after a manual stimulus were considered moving, and dead animals and animals without crawling behavior were considered not moving. Three different experiments with 60 worms each were performed.

### Pharyngeal and motility assays

The nematodes were synchronized by egg laying and cultured on NGM plates seeded with OP50 *E. coli* for food at 20°C. From the first to the eighth day of adulthood, the pharyngeal pumping rate, obtained by counting the number of times the terminal bulb of the pharynx contracts in a 1-min interval (pumps/min) (Diomede et al., 2014), and the motility test, based on counting the number of body movements in liquid (body bending/min) in a 1-min interval (Zanier et al., 2021), were performed.

### Electropharyngeograms

The nematodes were synchronized by egg laying and cultured on NGM plates seeded with OP50 *E. coli* for food at 20°C. On the first day of adulthood, they were collected in M9 buffer, settled by gravity and washed with M9 buffer to eliminate bacteria. They were transferred to a reaction tube, washed three times with M9 buffer and incubated in a 10 mM serotonin solution (Sigma-Aldrich) for 30 min. A ScreenChip™ System (InVivo Biosystems) was employed to record the voltage changes caused by the contraction of the pharynx in real-time, producing the electropharyngeograms (Lockery et al., 2012). Worms were loaded on a ScreenChip SC40 (InVivo Biosystems) with a syringe (0.01-1 ml), and the electropharyngeograms of single worms were recorded for ∼2 min. Only worms that showed pumping activity were recorded; those without pumping activity were discarded. The software programs NemAcquire 2.1 and NemAnalysis 0.2 were used for recording and analysis, respectively. The following parameters were measured: pump frequency, spike amplitude ratios, pump duration and IPI (Lockery et al., 2012).

### Mitochondrial ROS production

Mitochondrial ROS production was detected in live worms using MitoSOX™ Red staining (Thermo Fisher Scientific, Milan, Italy). The nematodes were synchronized by egg laying and cultivated on NGM plates until the L4 larval stage. Sixty worms were transferred onto freshly prepared 6 cm NGM plates containing 10 µM MitoSOX™ Red and seeded with UV-killed OP50 *E. coli* bacteria. After incubating for 16 h in the dark at 20°C, when worms were on the first day of adulthood, they were transferred for 1 h to new NGM plates spread with live OP50 *E. coli* bacteria to remove residual dye from the gut. As positive control, MNV worms at the L4 larval stage were incubated for 2 h with 0.5 mM H_2_O_2_, plated onto 6 cm NGM plates containing 10 µM MitoSOX™ Red and processed as described before.

For imaging, nematodes were mounted onto 2% agarose pad slides, anesthetized by adding 10 mM levamisole (Sigma-Aldrich) and fixed with ProLong™ Glass Antifade Mountant (Thermo Fisher Scientific). Images were acquired immediately with an inverted fluorescent microscope (IX-71, Olympus, Tokyo, Japan) equipped with a CCD camera. Pictures of the pharynx and body-wall muscles were obtained at 40× magnification with a TRITC filter set (Olympus), and the integrated intensity was calculated using Fiji's imaging software (Brinkmann et al., 2022).

### Fluorescent and confocal microscopy

Age-synchronized MNH::mCherry worms were picked onto 2% agar pads, anesthetized with 10 mM levamisole and fixed with ProLong™ Glass Antifade Mountant on the first day of adulthood. Images were acquired with an inverted fluorescent microscope (IX-71 Olympus) equipped with a CCD camera. Pictures of the pharynges were taken at 40× magnification with a TRITC filter set (Olympus). Images were also acquired by confocal microscopy using Nikon A1 confocal microscopes.

### TEM

From each sample, one semithin (1 μm) section was cut with a Leica EM UC6 ultramicrotome and mounted on glass slides for light microscopic inspection. Ultrathin (60-80 nm thick) sections of areas of interest were obtained, counterstained with uranyl acetate and lead citrate, and examined with an Energy Filter TEM (ZEISS LIBRA^®^ 120) equipped with a YAG scintillator slow-scan CCD camera.

### Pharmacological studies

Pharmacological studies were performed as previously described by our group (Diomede et al., 2014, 2017). On the first day of adulthood, age-synchronized MNH and MNV worms were collected, centrifuged (100 ***g*** for 3 min) and washed twice with M9 buffer. The nematodes (100 worms/100 μl) were treated for 2 h in 1.5 ml tubes with 0-1 mM ascorbic acid (Sigma-Aldrich), 0-200 μM doxycycline hydrochloride (Sigma-Aldrich) and 0-2 nM PBT2 (Prana Biotechnology, Parkville, Australia) (Diomede et al., 2017). Using the same protocol, worms were treated for 2 h with 10 mM PBS, pH 7.4 (100 worms/100 μl), as control (vehicle). At the end of incubation, worms were transferred to NGM plates seeded with OP50 *E. coli* bacteria and spotted with 0-1 mM ascorbic acid, 0-200 μM doxycycline or 0-2 nM PBT2. The pumping rate was measured 20 h later, as described above.

### Statistical analysis

No randomization was required for *C. elegans* experiments. All evaluations were done unaware of sample identity and treatment group. The data were analyzed using GraphPad Prism 9.4.1 software by unpaired, two-tailed Student's *t*-test, one-way or two-way ANOVA, and Bonferroni's post hoc test. IC_50_ values were determined using the same software. *P*<0.05 was considered significant. For lifespan and healthspan studies, the number of dead and censored animals was used for survival analysis in OASIS 2 (Han et al., 2016). *P*-values were calculated using the log-rank and Bonferroni's post hoc test between the pooled populations of animals.

## Supplementary Material

10.1242/dmm.052230_sup1 Supplementary information
