# Supplementary material for: Modeling immunoglobulin light chain amyloidosis in Caenorhabditis elegans
Source: Dis Model Mech. 2025 Jul 25;18(7):dmm052230. doi: 10.1242/dmm.052230 (PMC12320968; doi:10.1242/dmm.052230)
Supplement: Supplementary information [file dmm-18-052230-s1.pdf]

H7 Sequence

ATG**ACCTGCTCCCCTCTCCTCCTCACCCTCCTTATTCACTGCACCGGATCCTGGAC**
CCAATCCGTCCTCACCCAACCACCATCCGTCTCCGCCGCCCCAGGACAAAAGGTCACCA
TCTCCTGCTCCAACGTCGGAAAGAACTTCGTCTCCTGGTACCAACAATTCCCAGGAACC
GCCCCAAAGGTCGTCATCTACGACACCGACAAGCGTCCATCCGACATCCCAGACCGTTT
CTCCGGATCCAAGTCCGGAACCTCCGCCACCCTCGACATCACCGGACTCCAAACCGGA
GACGAGGCCGACTACTACTGCGGAACCTGGGACTCCGGACTCAACGGAGGAGTCTTCG
GAGGAGGAACCAAG**gtaagtttaaacatatatatactaactaaccctgattatttaaattttcag**GTCACCGTCCTC
GGACAACCAAAGGCCGCCCCATCCGTCACCCTCTTCCCACCATCCTCCGAGGAGCTCCA
AGCCAACAAGGCCACCCTCGTCTGCCTCATCTCCGACTTCTACCCAGGAGCCGTCACCG
TCGCCTGGAAGGCCGACTCCTCCCCAGTCAAGGCCGGAGTCGAGACCACCACCCCATC
CAAGCAATCCAACAACAAGTACGCCGCCTCCTCCTACCTCTCCCTCACCCCAGAGCAAT
GGAAGTCCCACAAGTCCTACTCCTGCCAAGTCACCCACGAGGGATCCACCGTCGAGAA
GACCGTCGCCCCAACCGAGTGCTCCTAA

M7 sequence

ATG**GCTTGGACTCCTTTATGGCTCACTCTCCTTACGCTGTGCATCGGATCCGTCGT**
CTCCTCCGAGCTCACCCAAGACCCAGCCGTCTCCGTCGCCCTCGGACAAACCGTCAAG
ATCACCTGCCAAGGAGACTCCCTCCGTATGTACTACGCCTCCTGGTACCAACAAAAGCC
AGCCCAAGCCCCAGTCCTCGTCATCTACGCCGAGAAGAACCGTCCATCCGGAATCCCAG
ACCGTTTCTCCGCCTCCTCCTCCGGATCCACCGCCTCCCTCACCATCACCGGAGCCCAA
GCCGAGGACGAGGCCGACTACTACTGCAACTCCCGTGACAACTCCGGAGACCACCTCG
TCTTCGGAGGAGGAACCAAGCTCACCGTCCTCGGACAACCAAAG**gtaagtttaaacatatatatac**
taactaaccctgattatttaaattttcagGCCGCCCCATCCGTCACCCTCTTCCCACCATCCTCCGAGGA
GCTCCAAGCCAACAAGGCCACCCTCGTCTGCCTCATCTCCGACTTCTACCCAGGAGCCG
TCACCGTCGCCTGGAAGGCCGACTCCTCCCCAGTCAAGGCCGGAGTCGAGACCACCAC
CCCATCCAAGCAATCCAACAACAAGTACGCCGCCTCCTCCTACCTCTCCCTCACCCCAG
AGCAATGGAAGTCCCACCGTTCCTACTCCTGCCAAGTCACCCACGAGGGATCCACCGTC
GAGAAGACCGTCGCCCCAACCGAGTGCTCCTAA

Fig. S1. The DNA sequence of the amyloidogenic cardiotoxic H7 LC and the non-amyloidogenic myeloma-derived M7 LC optimized to be expressed in *C. elegans*.

The human DNA secretion sequence is reported in red, and the intron sequence is written in blue.

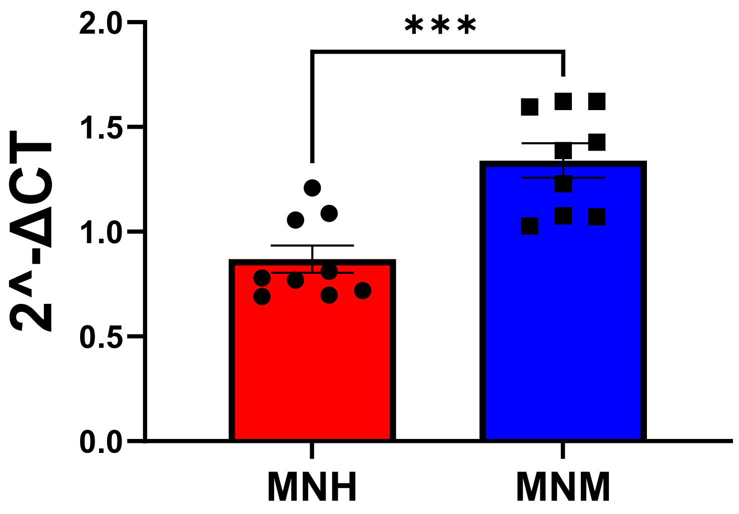

Fig. S2. Messenger RNA levels in transgenic worms

Quantitative real-time PCR analysis of mRNA levels in synchronized nematodes on the first day of adulthood. Specific primers recognizing the constant LC region were used. LC gene expression levels were compared to *cdc-42* housekeeping mRNA. Data are the mean ± SEM (n=9, from 3 different biological samples). ****p*<0.001 according to the Student t-test.

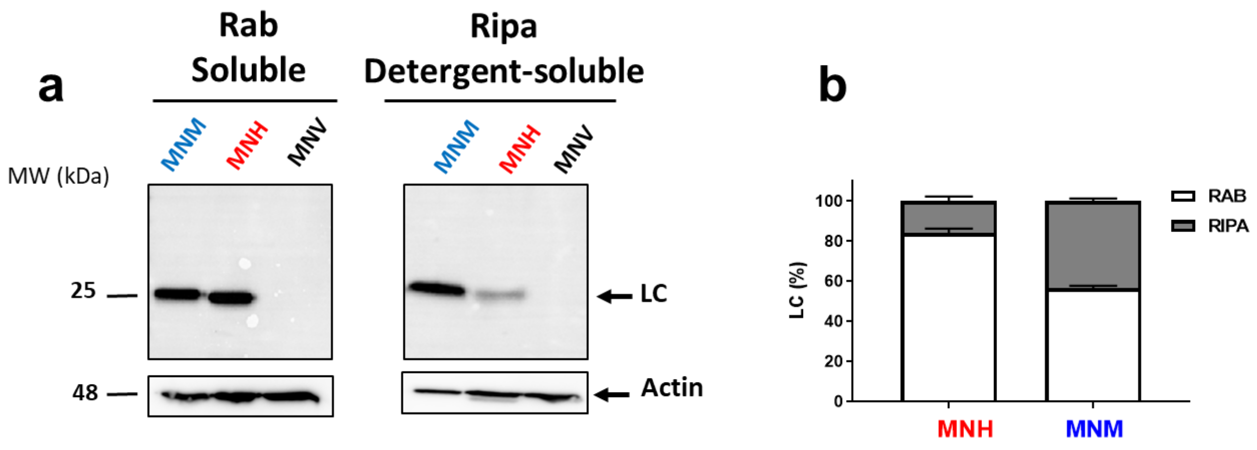

Fig. S3. Levels of the RAB soluble and RIPA detergent-soluble LCs

(**a**) RAB soluble and RIPA detergent-soluble fractions from the equivalent amounts of proteins from worms on the first day of adulthood were analyzed by immunoblotting using the anti-LC antibody (1:1000 dilution) or anti-actin antibody (1:2000 dilution). (**b**) LC/actin immunoreactivity in each fraction was determined and expressed as a percentage of the total LC. Data are mean ± SD (n = 3 from 2 assays). The LC percentage in RAB soluble fraction was higher in MNH worms (84%) compared to MNM (56%). The percentage of LC in the RIPA fraction was 16% and 43% in MNH and MNM, respectively.

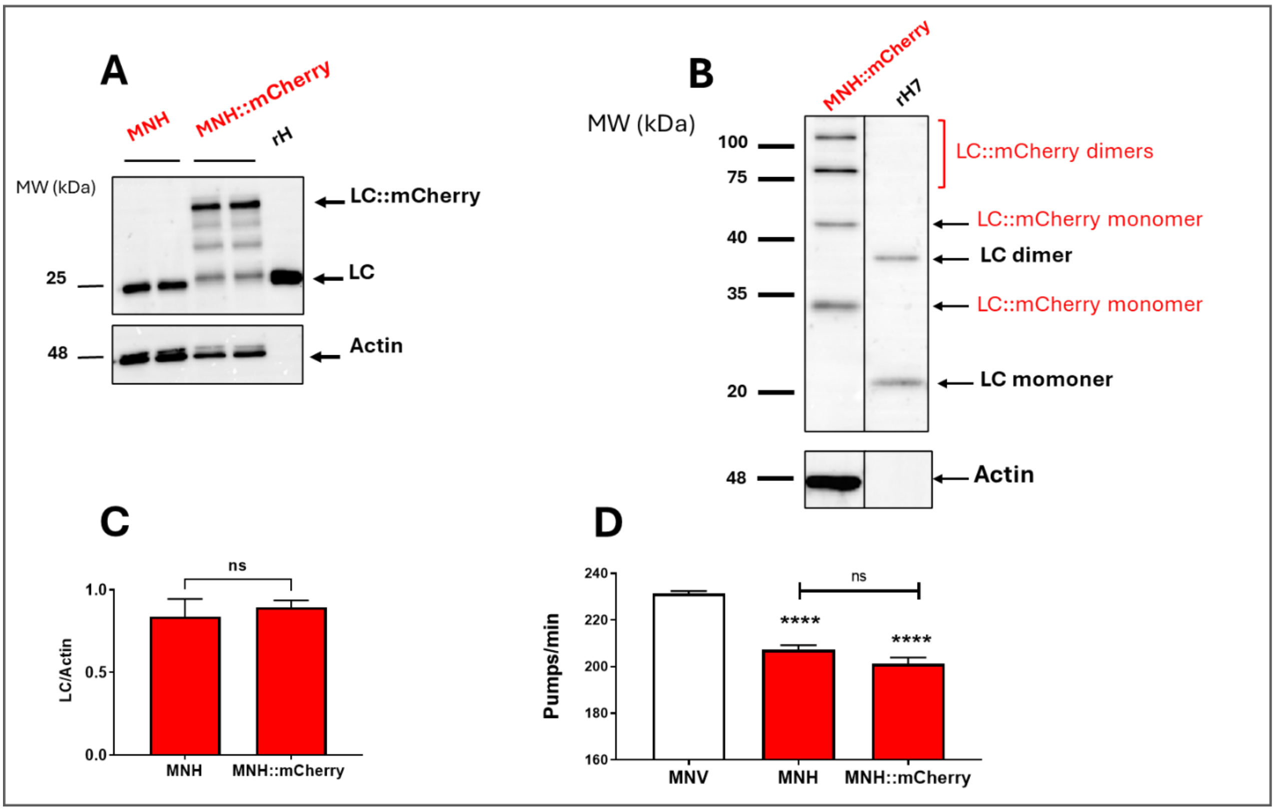

Fig. S4. Characterization of the transgenic MNH::mCherry *C. elegans* strain.
(**a, b**) Representative Western blot obtained under (**a**) reducing and (**b**) non-reducing conditions of LC in lysates of MNH and MNH::mCherry worms on the first day of adulthood. An equal amount of proteins (25 μg) was loaded in each gel lane and immunoblotted with anti-human λ total LC or anti-actin antibody. Recombinant H7 LC (rH, 50 ng) was loaded as a control. (**b**) Two monomeric LC::mCherry bands (produced by LC fused with the two fragments of mCherry under non-reducing conditions, of 11 or 30 kDa) and two bands in their dimeric forms are shown in red (Hutson et al., 2014). (**c**) Quantification of total LC expressed as the mean volume of the anti-human λ total LC band immunoreactivity of the Western blot in the (a)/actin band. Data are mean ± SEM (n = 3, from 2 independent experiments). No statistical difference (Student's t-test, p= 0.1997) was found between MNH and MNH::mCherry strains. (**d**) The pharyngeal activity of worms on the first day of adulthood is expressed as pumps/min. Data are the mean ± SEM (n = 30 worms/assay, four assays). **** p<0.0001 *vs.* MNV, one-way ANOVA, and Bonferroni's *post hoc* test.

Disease Models & Mechanisms • Supplementary information

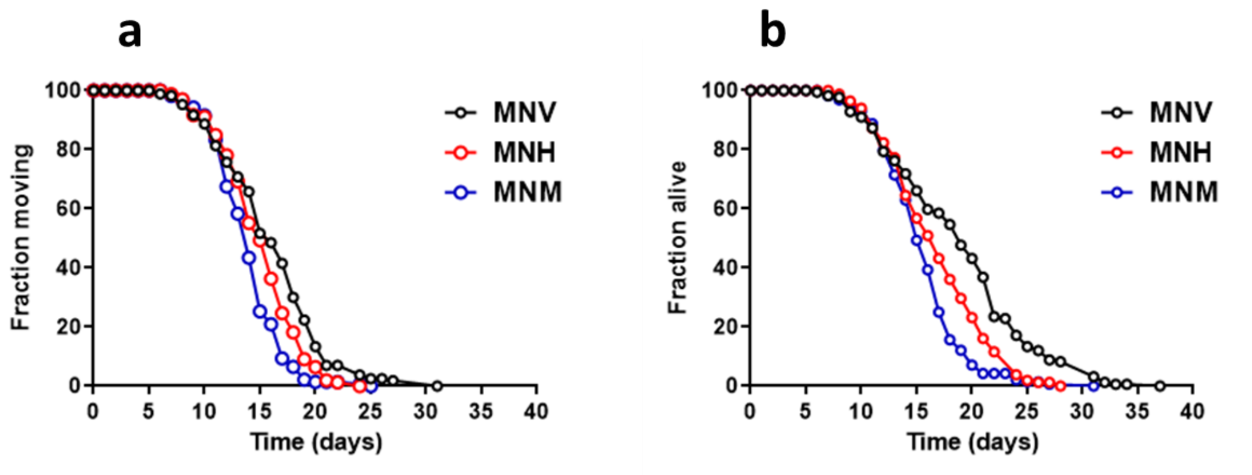

Fig. S5. Health span and lifespan of transgenic worms

(**a**) Health span and (**b**) lifespan curves of MNV, MNH, and MNM nematodes. Dead, alive, and censored animals were scored. Data are the mean ± SEM (n=180 worms from 3 independent experiments). See Supplemental Table 3 for mean lifespan, health span, and statistical analyses.

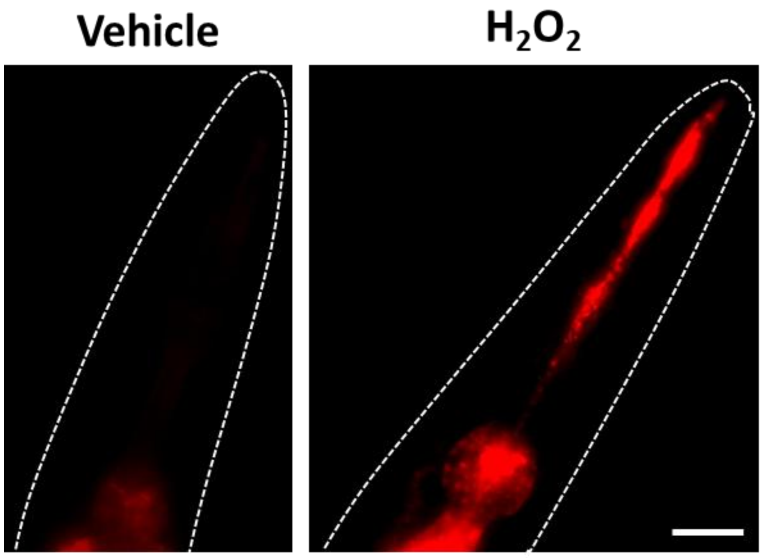

Fig. S6. Effect of hydrogen peroxide on mitochondrial superoxide production. Representative images of mitochondrial superoxide production in the pharynx of MNV worms administered with 10 mM PBS (Vehicle) or 0.5 mM H_2O_2 (H_2O_2), detected with MitoSOX™ Red. Scale bar = 50 µm.

Disease Models & Mechanisms • Supplementary information

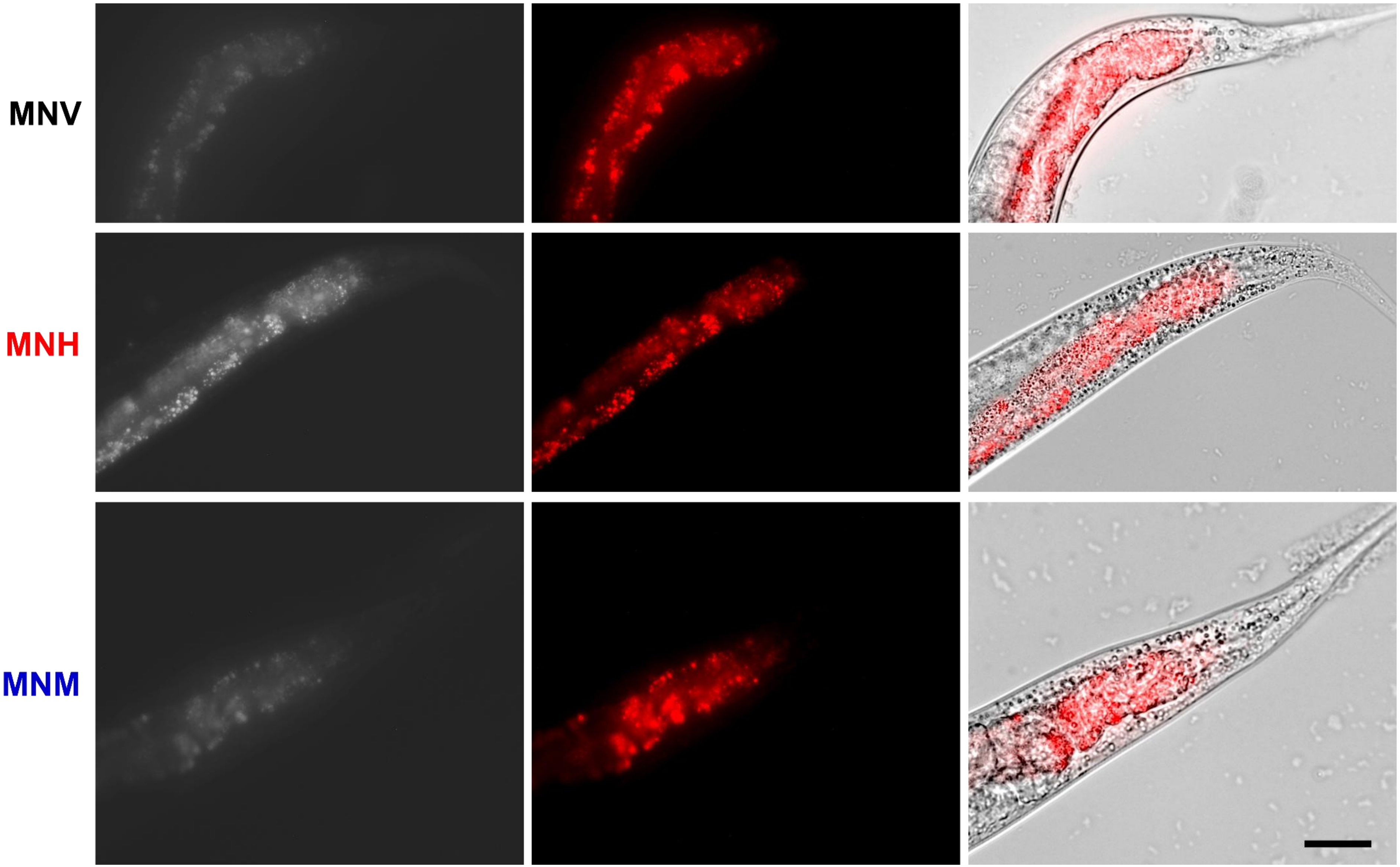

Fig. S7. Mitochondrial superoxide production in the body-wall muscle of transgenic worms. Representative images of mitochondrial superoxide production in the body-wall muscles of MNV, MNH, and MNM worms detected with MitoSOX™ Red. Scale bar = 50 µm.

Table S1. Clinical and biochemical characteristics of patients suffering from AL amyloidosis or multiple myeloma from which the sequence of LCs was deduced.

					**Biochemical characteristics**				**Cardiac parameters**				
Protein code	*Gender, age*	*Cardiac stage* [a]	*Diagnosis*	*Organ involved* [b]	*Germline gene*	*Deduced MW (kDa/pI)*	*Serum λ FLC (mg/L)*	*κ/λ FLC ratio*	*NT-proBNP (ng/L)*	*cTnl (ng/nl)*	*IVS (mm)*	*PW (mm)*	*EF (%)*
H7	M, 45	III	AL	Heart	IGLV1-51	22.3/6.15	477	0.01	8882	0.16	19	19	45
M7	M, 48		MM	-	IGLV2-23	22.8/8.20	573	0.01	14.5	0.003	10	10.5	67

Modified from (Diomede et al., 2017).
Reference ranges: serum *λ* FLC < 26.3 mg/L, *κ/λ* ratio 0.26–1.65; NT-proBNP <332 ng/L; cTnI <0.04 ng/ml.
[a] According to (Gertz et al., 2005). According to the International Consensus Panel criteria (Gertz et al., 2005).
AL, immunoglobulin light chain amyloidosis; cTnI, cardiac troponin I; EF, ejection fraction; FLC, free light chains; IVS, interventricular septum; M, male; MM, multiple myeloma; NT-proBNP, N-terminal prohormone of brain natriuretic peptide; pI, isoelectric point; PW, posterior wall.

Table S2. Summary of lifespan and health span analysis.

	Strain	MEAN (Days ± SEM)	p-value *vs* MNV	p-value *vs* MNH	No. of subjects	Censor
**Lifespan**	**MNV**	18.9 ± 0.5		0.0001	180	21
	**MNH**	16.7± 0.3	0.0001		180	23
	**MNM**	15.6± 0.3	<0.0001	0.0044	180	38
**Healthspan**	**MNV**	16.1± 0.4		0.0081	180	21
	**MNH**	15.1± 0.3	0.0081		180	23
	**MNM**	14.0± 0.2	<0.0001	0.0016	180	38

The *p*-values were calculated using the log-rank and Bonferroni's *post hoc* test between the pooled animal populations. The data are from three independent experiments with 60 worms each.

Table S3. List of the strains used in this study.

Strain name abbreviation	Strain	Genotype
MNH	COP2040 - knuSi825	*[pNU2100 (myo-3::H7::tbb-2u in ttTi5605,unc-119(+))] II ; unc-119(ed3) III*
MNM	COP2177 - knuSi837	*[pNU2101(myo-3::M7::tbb-2u in ttTi5605, unc-119(+))] II; unc-119(ed3) III*
MNV	COP2043 - knuSi827	*[pNU936(empty ttTi5605, unc-119(+))] II ; unc-119(ed3) III*
MNH::mCherry	COP2459 - knuls85	*[myo-3p::H7::mCherry::tbb-2 3'-UTR * knuSi825)] II*

Table S4. List of the primers used in this study.

**Name**	**Sequence (5'-3')**
H7/M7-Constant region- Forward	5'-CTCTTCCCACCATCCTCC-3'
H7/M7-Constant region -Reverse	5'-CGGCGTACTTGTTGTTGGAT-3'
cdc-42 -Forward	5'-CTGTTGTGGTGGGTCGAGAG-3'
cdc-42 -Reverse	5'-GTTGACGCAGAAGGGACTGA-3'
Y45F10D.4 -Forward	5'-ATCTTCCCTGGCAACCGAAT-3'
Y45F10D.4 -Reverse	5'-TGGGCGAGCATTGAACAGT-3'

Disease Models & Mechanisms • Supplementary information

References

Diomede, L., Romeo, M., Rognoni, P., Beeg, M., Foray, C., Ghibaudi, E., Palladini, G., Cherny, R. A., Verga, L., Capello, G. L., et al. (2017). Cardiac Light Chain Amyloidosis: The Role of Metal Ions in Oxidative Stress and Mitochondrial Damage. *Antioxid. Redox Signal.* 27, 567–582.

Gertz, M. A., Comenzo, R., Falk, R. H., Fermand, J. P., Hazenberg, B. P., Hawkins, P. N., Merlini, G., Moreau, P., Ronco, P., Sanchorawala, V., et al. (2005). Definition of organ involvement and treatment response in immunoglobulin light chain amyloidosis (AL): a consensus opinion from the 10th International Symposium on Amyloid and Amyloidosis, Tours, France, 18-22 April 2004. *Am J Hematol* 79, 319–328.

Hutson, T. H., Kathe, C., Menezes, S. C., Rooney, M.-C., Bueler, H. and Moon, L. D. F. (2014). The use of an adeno-associated viral vector for efficient bicistronic expression of two genes in the central nervous system. *Methods Mol Biol* 1162, 189–207.
